# Effects of Atomoxetine on Motor and Cognitive Behaviors and Brain Electrophysiological Activity of Dopamine Transporter Knockout Rats

**DOI:** 10.3390/biom12101484

**Published:** 2022-10-14

**Authors:** Maria Ptukha, Zoia Fesenko, Anastasia Belskaya, Arina Gromova, Arseniy Pelevin, Natalia Kurzina, Raul R. Gainetdinov, Anna Volnova

**Affiliations:** 1 Institute of Translational Biomedicine, Saint Petersburg State University, 199034 Saint Petersburg, Russia; 2 Faculty of Biology, Saint Petersburg State University, 199034 Saint Petersburg, Russia; 3 Saint Petersburg State University Hospital, Saint Petersburg State University, 199034 Saint Petersburg, Russia

**Keywords:** dopamine transporter knockout (DAT-KO) rats, ADHD, prepulse inhibition, power spectra, coherence, dopamine, norepinephrine transporter (NET), atomoxetine

## Abstract

Changes in dopaminergic and noradrenergic transmission are considered to be the underlying cause of attention deficit and hyperactivity disorder (ADHD). Atomoxetine (ATX) is a selective norepinephrine transporter (NET) inhibitor that is currently used for ADHD treatment. In this study, we aimed to evaluate the effect of atomoxetine on the behavior and brain activity of dopamine transporter knockout (DAT-KO) rats, which are characterized by an ADHD-like behavioral phenotype. Prepulse inhibition (PPI) was assessed in DAT-KO and wild type rats after saline and ATX injections, as well as behavioral parameters in the Hebb–Williams maze and power spectra and coherence of electrophysiological activity. DAT-KO rats demonstrated a pronounced behavioral and electrophysiological phenotype, characterized by hyperactivity, increased number of errors in the maze, repetitive behaviors and disrupted PPI, changes in cortical and striatal power spectra and interareal coherence. Atomoxetine significantly improved PPI and decreased repetitive behaviors in DAT-KO rats and influenced behavior of wild-type rats. ATX also led to significant changes in power spectra and coherence of DAT-KO and wild type rats. Assessment of noradrenergic modulation effects in DAT-KO provides insight into the intricate interplay of monoaminergic systems, although further research is still required to fully understand the complexity of this interaction.

## 1. Introduction

Dopamine (DA) and norepinephrine (NE) are neurotransmitters that are involved in the regulation of a wide range of brain functions. They are involved in sensory information processing, movement planning, plasticity, motivation and memory formation [1,2]. The two catecholaminergic systems are functionally and structurally interconnected in their biosynthesis, signaling pathways, areas of innervation and reuptake mechanisms [1,3,4,5]. In this regard, assessing the independent effects of DA and NE can be quite challenging, while the understanding of the interplay of these systems is crucial for the treatment of multiple disorders, including schizophrenia, depression, bipolar disorder, addiction and ADHD [1,6,7].

Attention deficit and hyperactivity disorder (ADHD) is a heterogeneous disorder, the etiology of which is still not fully understood. Symptoms include hyperactivity, impulsivity, inattention, lack of motivation and learning difficulties [8,9]. In ADHD, changes in DA and NE are thought to play a crucial role in the pathology of the disorder [10,11,12], although the contribution of serotonin system to at least some behavioral manifestations cannot be excluded [13,14]. One of the approved medications with proven clinical efficacy in reducing ADHD symptoms is atomoxetine (ATX, atomoxetine, LY139603) [15,16,17]. ATX is a selective norepinephrine transporter (NET) inhibitor that increases extracellular levels of NE, as well as DA, in NET-enriched areas, such as the PFC, the occipital cortex, the hypothalamus, the hippocampus and the cerebellum [18,19,20,21]. Unlike psychostimulant drugs used in pharmacotherapy of ADHD, ATX is not addictive and does not pose any risks for patients with comorbid anxiety or tics [15].

In this study, experiments were carried out on DAT-KO (dopamine transporter knockout) rats. The lack of dopamine transporter (DAT) results in an eight-fold increase in the level of extracellular dopamine in the striatum of DAT-KO rats compared to wild type (WT) [22]. A prominent behavioral phenotype is a result of such dramatic changes in dopaminergic signaling. DAT-KO rats exhibit hyperactivity, stereotypy, behavioral rigidity, deficiencies in motivation, learning and spatial orientation [22,23,24]. Due to these behavioral abnormalities and changes in the dopaminergic system, DAT-KO rats have been proposed as an animal model of ADHD [22,23]. The complete lack of DAT in these rats, as previously shown in mice [25], makes them an excellent model of persistent hyperdopaminergia, which can be used as a tool to study the mechanisms of other dopamine-associated diseases, such as schizophrenia, bipolar disorder, addiction, Huntington’s disease, Parkinson’s disease, etc.

This work is aimed at assessing the role of noradrenergic modulation in different dopaminergic conditions in the regulation of behavior and brain activity through ATX-mediated inhibition of NET in DAT-KO and DAT-WT rats. Involuntary attention, learning, spatial orientation and locomotor activity were evaluated in the Hebb–Williams maze and with PPI testing. Spectral power and interareal coherence of the PFC, striatum and motor cortex were analyzed.

## 2. Materials and Methods

### 2.1. Animals

A total of 17 DAT-KO and 17 WT males of the same age (3–4 months) were used in the experiments. Genotyping was performed according to a protocol described previously [22]. All experimental procedures were conducted in compliance with requirements regarding the care and treatment of laboratory animals and the Ethics committee of Saint Petersburg State University, St. Petersburg, Russia, resolution No. 131-03-10 of 22 November 2021. Before the experiments, rats were maintained in IVC cages (RAIR IsoSystem World Cage 500; Lab Products, Inc., Seaford, DE, USA) with free access to food and water, at a temperature of 22 ± 1 degrees C, 50–70% relative humidity and a 12 h light/dark cycle (light from 9 am). Experiments were carried out between 2 pm and 6 pm.

### 2.2. Hebb–Williams Maze

#### 2.2.1. Animals

Experiments were conducted on 10 DAT-KO and 10 WT adult male rats. For 5 days before the training, rats received food at a ratio of 90% of their regular diet (BioPro, Novosibirsk, Russia). Each animal was weighed daily prior to the experiment during all the experiments’ duration. 

#### 2.2.2. Experimental Setup

**Figure 1:**
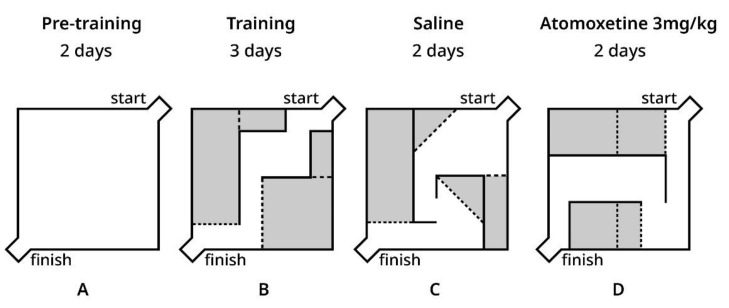
Layouts of the Hebb–Williams maze arenas used in experiments. (**A**) Empty arena used for familiarization during the pre-training period; (**B**) Layout used for the training period; (**C**) Layout used after saline injections; (**D**) Layout used after ATX injections. Walls indicated in black; error zones indicated in grey with dashed lines as the borders.

We used the Hebb–Williams maze to study animal spatial working memory. The Hebb–Williams maze consists of a square area, 75 × 75 × 25 cm. One corner is designated as the start of the maze, and the opposing corner contains a goal box with a food well for reward. Between trials, the maze surfaces were rubbed with peroxide solution. The behavioral task implied reaching the goal box to obtain food reinforcement. The interior walls of the maze are moveable, which allows the creation of new arena layouts and different routes through the maze (Figure 1). Each arena layout includes a correct way from start to finish and several dead ends, which are called “error zones”. Different arena layouts were used to diminish habituation’s influence on learning behavioral task rules. Each new arena was coupled with saline or ATX injections.

#### 2.2.3. Task Procedure

The pre-training period included a familiarization procedure in a maze with no walls during the first 2 days, and rats were allowed to individually explore the maze for 10 min (Figure 1A). Then, rats were trained in the same maze arena for 3 days (three trials for each animal per day) with reward only in a goal box food well for task rules acquisition (Figure 1B).

After these periods, rats received saline injections (0.9% NaCl, i.p. 30 min before testing) and were trained in the new arena configuration for 2 days (Figure 1C). After that, ATX (3 mg/kg i.p. 30 min before testing) was used for two consecutive days in a new arena configuration (Figure 1D). The behavioral variables such as the distance traveled, the time to reach the goal box, the number of entries into the error zones, time spent in the error zones and number of returns (a return was defined as a repeated visit of any given spot) were measured and analyzed by a video tracking system (EthoVision XT, Noldus Information Technology, Leesburg, VA, USA) with the video camera being placed above the maze.

### 2.3. PPI

Assessment of prepulse inhibition (PPI) was carried out on 20 adult male rats: DAT-KO (*n* = 10) and WT (*n* = 10).

The experimental setup consisted of a sound-attenuated chamber with four floor-mounted vibration sensors, Cambridge Electronic Design (CED, Cambridge, UK) Power1401-3A data acquisition interface, and Spike2 version 8 software (CED, Cambridge, UK). The movement of the animal was measured via floor-mounted vibrated sensors, the amplitude was converted to millivolts. Before the experiments, animals were presented with white noise (74 dB) for 20 min for habituation. The animals were tested two times, 2–3 days apart: 30 min after saline injection (0.9% NaCl i.p.) and 30 min after ATX injection (3 mg/kg i.p.).

On the day of the experiment, each animal was presented with “white noise” (74 dB, 10 min), followed by 10 prepulse stimuli (78 dB, 50 ms), then by 20 startle stimuli (100 dB, 50 ms), and finally, by 20 prepulse + startle combinations (100 ms delay between stimuli). The interval between stimuli and stimuli combinations varied from 10 to 14 s. Prepulse inhibition was calculated according to the following formula:

PPI = (1 − (prepulse + startle response amplitude)/startle response amplitude) × 100%

Video was recorded during the experiments in order to distinguish between spontaneous motor activity (seen mostly in DAT-KO rats) and startle responses.

### 2.4. ECoG and LFP Power Spectra and Coherence

Electrocorticogram (ECoG) and local field potential (LFP) recording was carried out on 14 adult male rats: DAT-KO (*n* = 7) and WT (*n* = 7).

#### 2.4.1. Electrode Placement

Epidural screws were used for ECoG recordings (1 mm in diameter; 1 mm in length; steel), while intracerebral electrodes were used for LFP recordings (50 μm in diameter; 2.5 mm/5 mm in length; tungsten wire in perfluoroalkoxy polymer isolation). For each animal, four electrodes were implanted under isoflurane: epidural reference electrode (7 mm posterior to bregma and 3 mm lateral to midline); primary motor cortex epidural electrode (1.5 mm posterior to bregma and 1.5 mm lateral to midline); prefrontal cortex (PFC) (2.5 mm in length, 2 mm anterior to bregma and 1 mm lateral to midline); striatal intracerebral electrode (5 mm in length, 0 mm from bregma and 3 mm lateral to midline). Correct electrode placement was achieved by use of a micromanipulator in a stereotaxic frame, in which the animal’s head was fixed during the whole operating procedure. Electrodes were fixed on the skull with dental cement.

#### 2.4.2. Electrocorticogram and Local Field Potential Recordings

Experimental setting for electrophysiological recordings consisted of an amplifier (×1000 gain), Cambridge Electronic Design (CED) Power1401-3A data acquisition interface, and Spike2 software (CED), sampling rate 25,000 Hz. During the recording process, animals were placed in 25 × 25 × 25 cm plexiglas box, which, along with the amplifier, was located in a Faraday cage.

Brain activity was recorded, on two different days, for an hour after saline injection (0.9% NaCl i.p., 30 min before recording) and for an hour after ATX injection (3 mg/kg i.p., 30 min before recording). For behavior monitoring, video was recorded simultaneously with brain activity. Only parts of recordings where the animals were awake were used in subsequent analysis.

#### 2.4.3. Data Analysis

Before the analysis, the sampling rate of the recordings was changed to 1000 Hz in order to increase the resolution of the resulting power spectra. For each recording, 200 s without artifacts were selected for analysis. Data were analyzed with a script (COHER.s2s, CED official website), which calculates the power spectra of each waveform signal through Fast Fourier Transform, as well as the coherence of two signals. A power spectrum is the frequency to power ratio of the signal, while coherence is a function of frequency, where for two waveforms to be completely coherent at a particular frequency over a given time range, the phase shift between the waveforms at that frequency must be constant, and the amplitudes of the waves at that frequency must have a constant ratio. The resulting data were in the 0.9–75 Hz range after data in the 0–0.8 range were excluded due to the abundance of artifacts. The following ranges for electroencephalographic rhythms were used for analysis and interpretation: delta (0.9–3 Hz), theta (4–8 Hz), alpha (9–11 Hz), lower beta (12–19 Hz), higher beta (20–29 Hz), lower gamma (30–48), and higher gamma (52–74 Hz).

### 2.5. Statistical Analysis

PPI and Hebb–Williams variables were compared with Brown–Forsythe and Welch ANOVA (one-way analysis of variance which does not assume equal variance) and Holm–Sidak’s multiple comparisons test. Before that, all data were analyzed for Gaussian distribution with D’Agostino–Pearson normality test. Power spectra and coherence were compared by bands with two-way ANOVA. All data are presented as mean ± SEM.

## 3. Results

### 3.1. Hebb–Williams Maze

During the experiments, we measured the following behavioral variables: distance traveled, time to reach the goal box, number of entries into the error zones, time spent in the error zones and number of returns. DAT-KO rats exhibited hyperactivity and stereotypy throughout all stages of the experiment, including the pre-training and training stages, which was described in a previous article [26]. 

**Figure 2:**
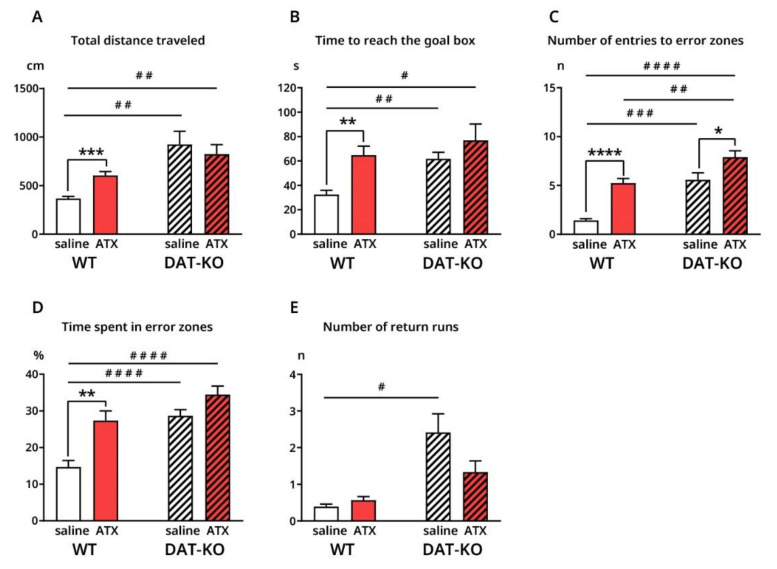
Behavioral characteristics of DAT-KO and WT rats after saline and ATX injections in Hebb–Williams maze (**A**–**E**). *, # *p* < 0.05; **, ## *p* < 0.01; ***, ### *p* < 0.001; ****, #### *p* < 0.0001; Brown–Forsythe and Welch ANOVA, Holm–Sidak’s multiple comparisons test.

After saline injections, DAT-KO demonstrated a poorer performance compared to WT, according to all analyzed variables. Total distance traveled by DAT-KO before reaching the goal box was significantly increased compared to WT (924.1 ± 134.8 cm vs. 367.1 ± 21.6 cm; *p* = 0.0074) (Figure 2A), as well as time required to reach the goal box (61.78 ± 5.4 s vs. 32.5 ± 3.5 s; *p* = 0.0014) (Figure 2B). DAT-KO rats demonstrated a significantly larger number of entries to the error zones (5.5 ± 0.7 vs. 1.4 ± 0.2; *p* = 0.001) (Figure 2C), along with an increased percentage of time spent in the error zones (14.7 ± 1.8 vs. 28.7 ± 1.7; *p* < 0.0001), compared to WT (Figure 2D). Finally, the number of returns was significantly greater in DAT-KO rats than in WT (2.4 ± 0.5 vs. 0.4 ± 0.1; *p* = 0.0129) (Figure 2E).

ATX injections majorly affected wild type animals. Total distance (604.7 ± 39.9 cm vs. 367.1 ± 21.6 cm; *p* = 0.0005) and time to reach the goal box (64.9 ± 7.3 s vs. 32.5 ± 3.5 s; *p* = 0.0045) significantly increased under ATX, compared to saline injections (Figure 2A,B). ATX had a similar effect on the number of visits (5.2 ± 0.5 vs. 1.4 ± 0.2; *p* < 0.0001) and percentage time spent in error zones (27.4 ± 2.6 vs. 14.7 ± 1.8; *p* = 0.003) (Figure 2C,D). In general, wild type rats demonstrated a poorer performance after ATX than after saline. 

Contrastingly, the only significant difference found in DAT-KO rats under ATX, was in the number of entries into the error zones, which was significantly higher compared to that after saline (7.9 ± 0.7 vs. 5.6 ± 0.7; *p* = 0.0293) (Figure 2C). The remaining characteristics seemed to improve in DAT-KO after ATX injections, although not significantly when compared to saline

After ATX injections, no significant differences were observed between DAT-KO and WT animals, except for the number of entries to error zones (7.9 ± 0.7 vs. 5.2 ± 0.5, *p* = 0.0097) (Figure 2C). It should also be noted that when compared to WT after saline, DAT-KO under ATX did not show a significant difference in the number of returns (Figure 2E), in contrast to other variables (Figure 2A–E).

### 3.2. PPI

The amplitude of the startle reflex and prepulse inhibition (PPI) index were measured in DAT-KO and WT rats.

**Figure 3:**
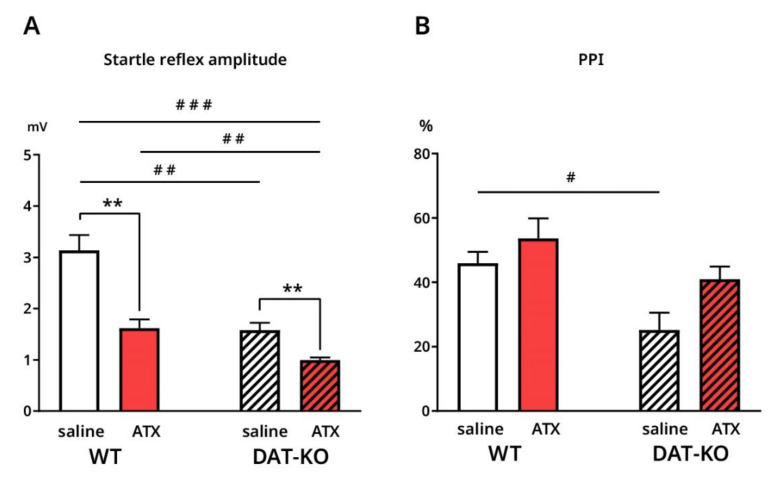
Amplitude of startle reflex and PPI index in DAT-KO and WT rats after saline and after ATX (**A**,**B**). # *p* < 0.05; **, ## *p* < 0.01; ### *p* < 0.001; Brown-Forsythe and Welch ANOVA, Holm–Sidak’s multiple comparisons test.

In DAT-KO, the amplitude of startle reflex was significantly smaller than in WT after saline injections (1.6 ± 0.1 mV vs. 3.1 ± 0.3 mV; *p* = 0.0017) (Figure 3A). After ATX injections, the amplitude of startle reflex was decreased, compared to saline, both in WT (1.6 ± 0.2 mV vs. 3.1 ± 0.3 mV; *p* = 0.0017) and in DAT-KO (0.99 ± 0.1 mV vs. 1.6 ± 0.1 mV; *p* = 0.0056) (Figure 3A). Under ATX startle reflex in DAT-KO was still lower when compared to WT (0.99 ± 0.1 mV vs. 1.6 ± 0.2 mV; *p* = 0.0056) (Figure 3A).

As for PPI, this variable was lower in DAT-KO after saline, compared to WT (25.2 ± 5.3 vs. 46 ± 3.5; *p* = 0.0303) (Figure 3B). ATX injections did not result in significant differences compared to saline in either of the groups (Figure 3B). However, we also compared PPI in DAT-KO after ATX to PPI in WT after saline to see if ATX had a positive effect on knockout animals, which brought their performance closer to control animals. As shown in Figure 3B, there is no significant difference between these PPI values, despite the significant difference in the amplitude of startle reflex (0.8 ± 0.5 mV vs. 3.1 ± 0.3 mV; *p* = 0.0002) (Figure 3A).

### 3.3. Power Spectra and Coherence of Brain Activity

Power spectra and coherence were analyzed via division of the range and comparison of electroencephalographic rhythms according to the following ranges: delta (0.9–3 Hz), theta (4–8 Hz), alpha (9–11 Hz), lower beta (12–19 Hz), higher beta (20–29 Hz), lower gamma (30–48), higher gamma (52–74 Hz). All p-values corresponding to each comparison can be found in Table S1 of Supplementary Materials, while most are presented in this section.

#### 3.3.1. Power Spectra and Coherence after Saline Injections

**Figure 4:**
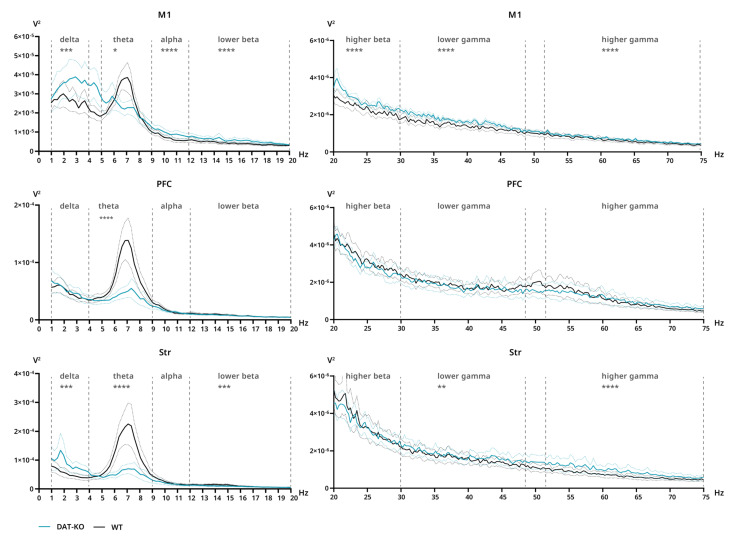
Power spectra of brain activity of DAT-KO (teal) and WT (black) rats recorded from motor cortex (M1), prefrontal cortex (PFC) and striatum (Str). 20–75 Hz range presented separately due to scale difference. Data are presented as mean (solid line) ± SEM (dotted line). Dashed lines represent electroencephalographic rhythms: delta (0.9–3 Hz), theta (4–8 Hz), alpha (9–11 Hz), lower beta (12–19 Hz), higher beta (20–29 Hz), lower gamma (30–48), higher gamma (52–74 Hz). The range of theta rhythm in M1 changed to 5–8 Hz. * *p* < 0.05; ** *p* < 0.01; *** *p* < 0.001; **** *p* < 0.0001; two-way ANOVA.

Power spectra and interareal coherence were evaluated in DAT-KO and WT rats after saline and ATX injections. After saline injections, significant differences in power were found in all three analyzed areas. The decreased power of DAT-KO brain activity compared to WT rats can be clearly seen in the theta range for all three areas (M1: *p* = 0.0327; PFC: *p* < 0.0001; Str: *p* < 0.0001) (Figure 4). Additionally, in M1 significant differences were found in the remaining bands, although in those cases power was increased in DAT-KO compared to WT (delta: *p* = 0.0026; alpha–higher gamma: *p* ≤ 0.0001) (Figure 4). In DAT-KO striatum, power was also higher than in WT in delta, lower beta and gamma + higher gamma ranges (delta and lower beta: *p* = 0.0006; lower gamma: *p* = 0.0063; higher gamma: *p* < 0.0001) (Figure 4).

**Figure 5:**
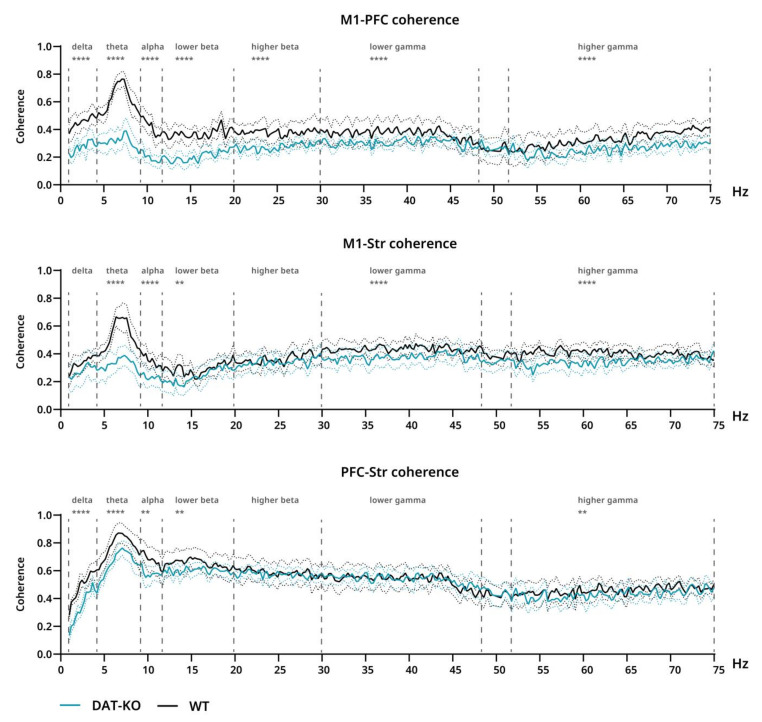
Coherence of brain activity of DAT-KO (teal) and WT (black) rats. Interareal coherence is presented for pairs of brain areas: motor cortex (M1), prefrontal cortex (PFC) and striatum (Str). Degree of coherence is expressed in fractions of one. Data are presented as mean (solid line) ± SEM (dotted line). Dashed lines represent electroencephalographic rhythms: delta (0.9–3 Hz), theta (4–8 Hz), alpha (9–11 Hz), lower beta (12–19 Hz), higher beta (20–29 Hz), lower gamma (30–48), higher gamma (52–74 Hz). ** *p* < 0.01; **** *p* < 0.0001; two-way ANOVA.

Significant differences were also found in coherence, which was generally lower in DAT-KO than in WT in all cases (Figure 5). In M1-PFC this effect can be seen throughout the whole range (all bands: *p* < 0.0001), while in M1-Str it encompasses all bands except delta and higher beta (lower beta: *p* = 0.0032; all other: *p* < 0.0001) and in PFC-Str–all except higher beta and lower gamma (delta, theta: *p* < 0.0001; alpha: *p* = 0.0042; lower beta: *p* = 0.0015; higher gamma: *p* = 0.0033) (Figure 5).

#### 3.3.2. Power Spectra and Coherence after Atomoxetine Injections

**Figure 6:**
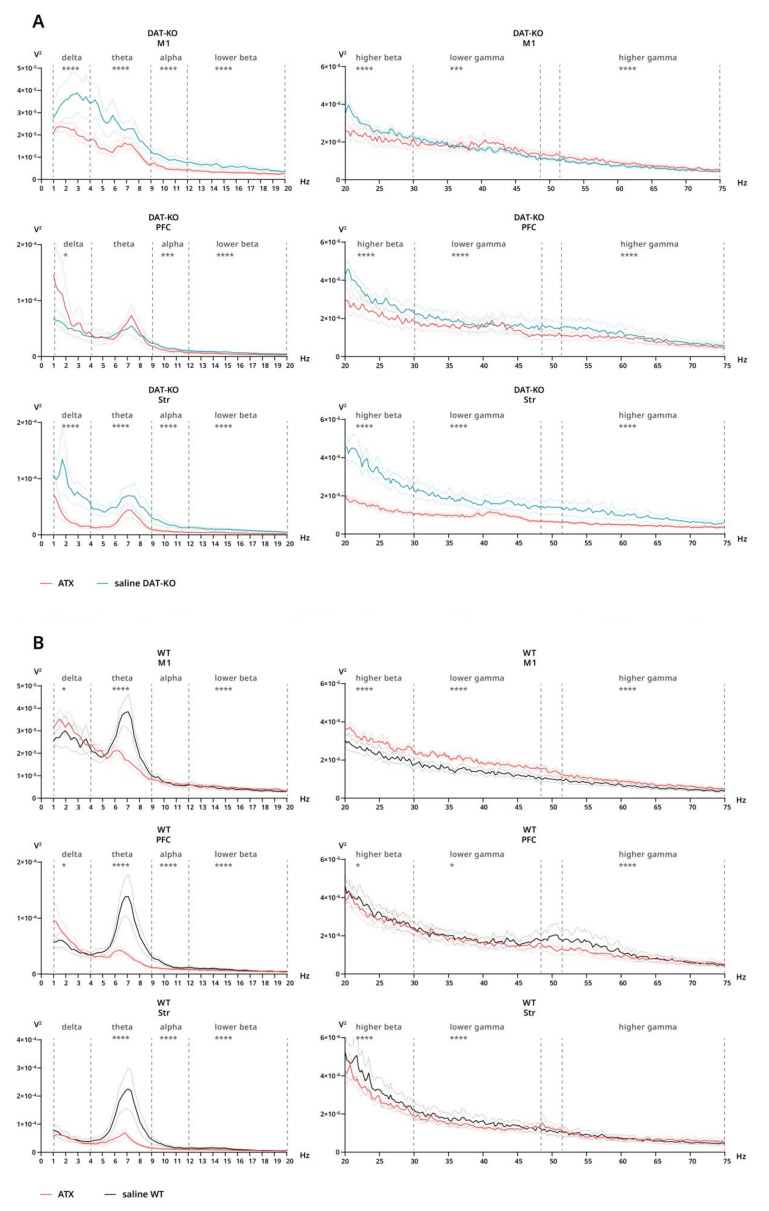
Power spectra of brain activity of DAT-KO and WT rats after saline and atomoxetine injections recorded from motor cortex (M1), prefrontal cortex (PFC) and striatum (Str). 20–75 Hz range presented separately due to scale difference. (**A**) Power after saline (teal) and ATX (red) in DAT-KO. (**B**) Power after saline (black) and ATX (red) in WT. Data are presented as mean (solid line) ± SEM (dotted line). Dashed lines represent electroencephalographic rhythms: delta (0.9–3 Hz), theta (4–8 Hz), alpha (9–11 Hz), lower beta (12–19 Hz), higher beta (20–29 Hz), lower gamma (30–48), higher gamma (52–74 Hz). * *p* < 0.05; *** *p* < 0.001; **** *p* < 0.0001; two-way ANOVA.

Under ATX, a general decrease in power can be seen in DAT-KO (Figure 6A). In the striatum, power is significantly decreased compared to saline throughout the whole range (all bands: *p* < 0.0001) (Figure 6A), while in M1 power is significantly lower after ATX in all bands (*p* < 0.0001) except lower and higher gamma, where power is significantly higher under ATX (lower gamma: *p* = 0.0003; higher gamma: *p* < 0.0001) (Figure 6A). In PFC, differences have not been found in the theta range, while in all other bands, the power is significantly decreased (alpha: *p* = 0.0008: lower beta—higher gamma: *p* < 0.0001), except delta, where the effect is the opposite (*p* = 0.0296) (Figure 6A).

In WT, under ATX, a prominent decrease can be seen in the theta range in all areas, compared to saline (*p* < 0.0001) (Figure 6B). Other than that, the opposite effect, although much less prominent, is seen in the delta range in M1 (*p* = 0.0498) and PFC (*p* = 0.0182) (Figure 6B). Curiously, while in M1 power under ATX is significantly higher in the higher beta–higher gamma range compared to saline (*p* < 0.0001) (Figure 6B), the opposite effect can be seen in the same bands in PFC (higher beta: *p* = 0.0402; lower gamma: *p* = 0.0105; higher gamma: *p* < 0.0001) and in higher beta—lower gamma bands in the striatum (*p* < 0.0001) (Figure 6B).

**Figure 7:**
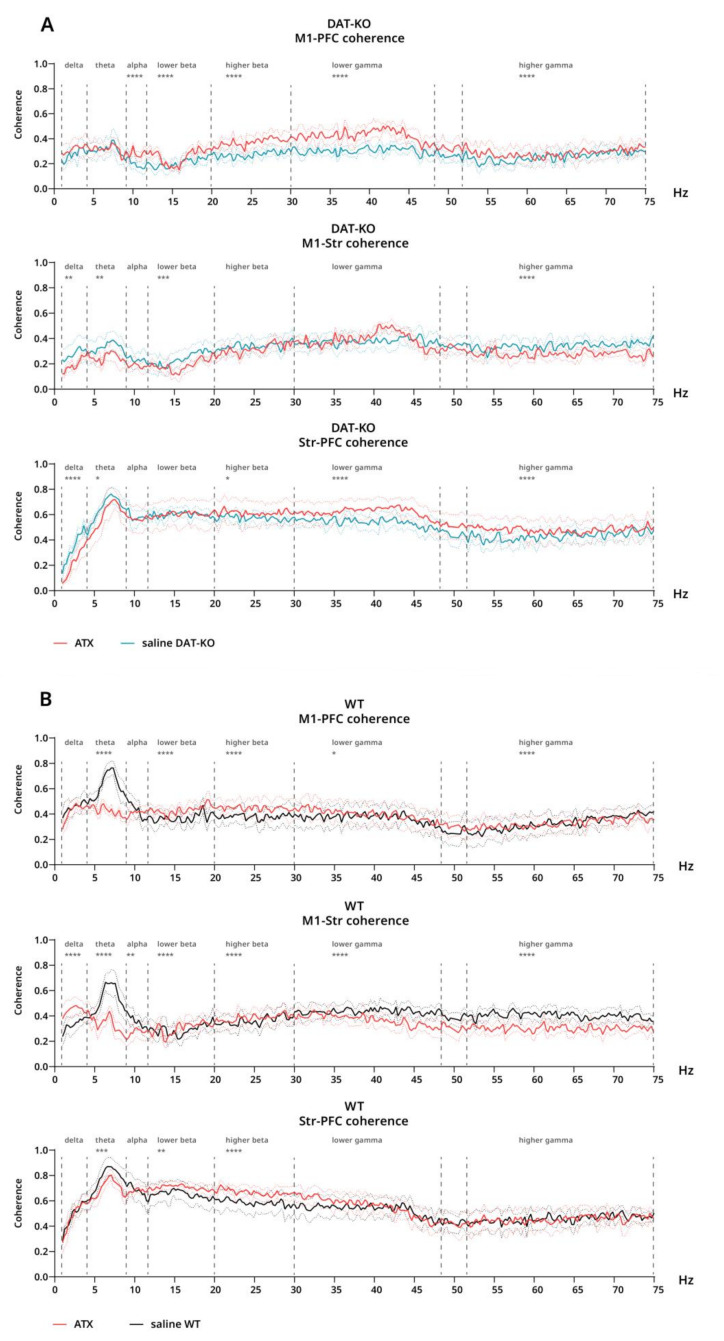
Coherence of brain activity of DAT-KO and WT rats after saline and atomoxetine injections. Interareal coherence is presented for pairs of brain areas: motor cortex (M1), prefrontal cortex (PFC) and striatum (Str). (**A**) Coherence after saline (teal) and ATX (red) in DAT-KO. (**B**) Coherence after saline (black) and ATX (red) in WT. Degree of coherence is expressed in fractions of one. Data are presented as mean (solid line) ± SEM (dotted line). Dashed lines represent electroencephalographic rhythms: delta (0.9–3 Hz), theta (4–8 Hz), alpha (9–11 Hz), lower beta (12–19 Hz), higher beta (20–29 Hz), lower gamma (30–48), higher gamma (52–74 Hz). * *p* < 0.05; ** *p* < 0.01; *** *p* < 0.001; **** *p* < 0.0001; two-way ANOVA.

For coherence under ATX compared to saline, opposing effects can be observed in different cases for DAT-KO. In M1-PFC, an increase can be seen in all bands except for delta and theta (alpha–lower gamma: *p* < 0.0001; higher gamma: *p* = 0.0003) (Figure 7A). Contrastingly, a significant decrease is seen in M1-Str in all bands except alpha and higher beta–lower gamma (delta: *p* = 0.0048; theta: *p* = 0.0016; lower beta: 0.0003; higher gamma: *p* < 0.0001) (Figure 7A). Additionally, in PFC-Str, coherence is decreased under ATX compared to saline in the delta (*p* < 0.0001) and theta (*p* = 0.011) bands, while in the whole gamma range, an opposite effect can be seen (*p* < 0.0001) (Figure 7A).

In WT rats, a significant decrease in coherence under ATX compared to saline can be seen in the theta range in all cases (*p* ≤ 0.0002) (Figure 7B). A decrease in coherence can also be observed throughout the whole gamma range in M1-Str (*p* < 0.0001), while in the delta band, there is an opposite effect (*p* < 0.0001) (Figure 7B). Finally, coherence is significantly increased in lower and higher beta bands both in M1-PFC and in PFC-Str under ATX when compared to saline (*p* ≤ 0.011) (Figure 7B).

## 4. Discussion

Due to the pronounced behavioral phenotype, DAT-KO rats have been proposed as an animal model of attention deficit and hyperactivity disorder (ADHD), following their well-studied mouse counterpart [22,26,27,28]. In this study, we investigated the behavior and electrophysiological parameters of DAT-KO rats under atomoxetine, which has been shown to improve all three major aspects of ADHD—hyperactivity, inattention and impulsivity [15,16]—through an increase in extracellular levels of DA and NE in NET enriched areas [29,30,31,32]. In rats, ATX has been repeatedly reported to decrease impulsivity [33,34,35,36]. Hyperactivity has been shown to be reduced by ATX in two other putative ADHD models—mice lacking pituitary adenylate cyclase-activating polypeptide and neurokinin-1 receptor ‘knockout’ mice [37,38]—while in spontaneously hypertensive rats (SHR), another putative ADHD model, results regarding impulsivity and inattention have been inconclusive [39].

In this study we demonstrate, not for the first time [22,40,41], the ADHD-like phenotype of DAT-KO rats: in the Hebb–Williams maze, compared to WT, DAT-KO rats made more errors, took a longer time and traveled a longer distance to reach the goal box and exhibited perseverative returns. Errors, time and distance are parameters that may be interpreted in different ways to indicate hyperactivity, impulsivity, deficient attention and working memory, while the number of return runs indicates repetitive (perseverative) behaviors of DAT-KO rats. We may hypothesize that most of these functions are in some way disrupted in DAT-KO since dopamine signaling plays a crucial role in the aforementioned processes and there is significant overlapping in encoding pathways [42,43]. Acute atomoxetine injection did not seem to majorly affect the performance of DAT-KO rats. Moreover, in wild type, ATX injection resulted in an overall decline in performance, including increased locomotor activity and number of errors.

Nevertheless, ATX injection led to a reduction in the number of return runs in DAT-KO, making this parameter indistinguishable from drug-naïve WT, which indicates that ATX influenced repetitive behavior. ATX has been proposed as treatment for several disorders characterized by repetitive behaviors, including not only ADHD, but also OCD (obsessive compulsive disorder) and hoarding disorder. ATX has been shown to reduce compulsivity and prevent its development in rats, as measured in schedule-induced polydipsia [44,45]. In a pre-clinical to clinical study, ATX was shown to reduce compulsive-like behaviors of mice in the marble burying test, as well as compulsive hoarding in humans [46]. In that study, ATX did not appear to affect locomotor activity or exploratory behaviors.

Prepulse inhibition, which is a measure of sensorimotor gating, was also tested in the present study to evaluate involuntary attention in DAT-KO rats. The testing revealed significantly lower amplitude of startle reflex in DAT-KO rats compared to WT, as well as a lower PPI index, which is in line with the previously observed PPI deficits in DAT-KO mice [47,48,49]. ATX injection led to normalization of PPI in DAT-KO, despite lower amplitude of startle reflex. Atomoxetine has been previously shown to improve PPI in mice with [38,50] and without PPI deficits [51], along with other NET blockers [49]. It should be noted that PPI has not been found to be impaired in ADHD [52,53], although there is conflicting evidence regarding P50—another measure of sensory gating, derived from EEG [54,55]. However, marked PPI deficits have been repeatedly shown in other disorders, including the aforementioned OCD [56,57] and schizophrenia [58,59].

Power spectra and interareal coherence of cortex and striatum of DAT-KO and WT rats were evaluated in this study. Various cognitive disorders are characterized by changes in brain activity that are associated with behavioral abnormalities. However, for many of those conditions, it has been challenging to distinguish specific biomarkers that can be derived from EEG. In ADHD, an increase in power in the theta range is the most prominent EEG feature, which has been reported for both children [60] and adults [61]. This feature has been proposed as a prominent biomarker, which can be used for ADHD diagnosis. In our study, DAT-KO rats, a putative animal model of ADHD, demonstrated quite the opposite—a prominent decrease in power in the theta range in PFC, motor cortex and striatum—while throughout the rest of the range, power was generally increased.

Acute ATX injection in WT led to a prominent decrease in power in the theta range in cortex and striatum, as well as an increase in delta and changes in beta and gamma ranges. Treatment with atomoxetine in ADHD patients has been reported to reduce posterior theta and increase beta activity after acute administration [62]. In long-term treatment, ATX was shown to normalize increases in alpha and delta ranges, which were characteristic for patients who responded to treatment, while those patients who displayed increases in beta and theta ranges did not respond to ATX [63]. In our case, DAT-KO animals displayed widely distributed decreases in power, especially in M1, that were somewhat corrected by ATX administration, which led to a general decrease observed in power spectra.

In addition to power spectra, coherence of EEG signal is an easily derived parameter that is a measure of functional synchronization between brain areas. In children with ADHD, increased frontal intra- and interhemispheric coherence in the theta band is the most consistently found characteristic, while reduced frontal gamma interhemispheric coherence has also been noted [64]. Meanwhile, we found DAT-KO rats to have generally lower coherence, when compared to WT. Somewhat similar data, however, have been reported for adults with ADHD: reduced alpha and delta coherence, as well as a lack of increase in theta coherence, which may be associated with hyperactivity—a symptom that typically reduces with increasing age [65], while alpha coherence deficit could be associated with inattention and can also be seen in children with ADHD [66].

In WT rats, ATX injection led to a significant decrease in coherence in the theta range in cortex and striatum. Additionally, in M1-PFC and PFC-Str in WT an increase in coherence is seen in the gamma range, while in M1-Str gamma-coherence is decreased. These changes could be seen as normalization when compared to the most frequently found coherence deficits in children increased theta and reduced gamma frontal coherence [64]. For DAT-KO, under ATX, M1-PFC coherence was generally increased and M1-striatum coherence showed a general decrease, while for coherence between PFC and striatum, ATX led to a decrease in the lower frequencies and an increase in the higher ones.

Despite the amount of research conducted on underlying mechanisms of ADHD, many aspects are still unclear, given the vast degree of interplay between monoaminergic systems. There is evidence that cortical dopamine, norepinephrine and serotonin play the central role in the pathology of the disorder [14,67,68], while recent findings suggest that the paradoxical calming effect of psychostimulants in ADHD, as well as in DAT-KO mice, is mediated predominantly by dopamine [13]. Deficits in DA transmission have been considered the main cause of other disorders, such as schizophrenia (SZ). The hyperactivity of subcortical DA transmission is thought to be responsible for the positive symptoms of SZ, while hypoactivity of the cortical DA is thought to be responsible for cognitive deficits [69]. Elevated DA in the striatum in DAT-KO may be the underlying cause for some abnormalities we observed in DAT-KO rats. In addition to PPI, which is disrupted in SZ patients and in DAT-KO rodents, electrophysiological biomarkers also point towards similar nature of behavioral deficits. A global decrease in theta power has been noted as one of the hallmark biomarkers of SZ as it has been reported for patients with both predominantly positive and predominantly negative symptoms [70]. An increase in delta power, especially frontally, is thought to be associated with negative symptoms of SZ [70,71]. Although an increase in delta cannot be seen in PFC in DAT-KO, it is significantly elevated in M1 and striatum, while theta is consistently decreased in DAT-KO compared to WT in cortex and striatum. Theta power is thought to underlie mental functions such as memory formation and retrieval, specifically encoding processes in working memory [72,73,74]. Deficits in working memory in DAT-KO rats have been reported before [22,24], as well as in this study, so a decrease in theta may be a correlate of these processes.

NET blockade has been shown to inhibit hyperactivity in DAT-KO mice [13,49] by nisoxetine and desipramine, see also [26]. In our study, atomoxetine failed to produce a similar result in DAT-KO rats. We assume that the dose of ATX used in our study (3 mg/kg), although sufficient to reduce hyperactivity in other mutant mouse models [38,39], was not enough to have that effect in DAT-KO rats.

However, in our study, ATX managed to correct PPI deficits and repetitive behavior in DAT-KO rats. Despite the previously explored assumption that it is DA that plays the main role in the effects of NET inhibition in DAT-KO and ADHD, we hypothesize that sensorimotor gating and repetitive behaviors are mediated by ATX mainly through NE. This assumption is based on our previous finding that guanfacine, an α2-adrenergic agonist, produced a very similar effect on PPI (unpublished data) and the number of perseverative returns [26], but did not affect hyperactivity or the number of errors (although the time spent in errors zones decreased) [26]. Therefore, our findings may indicate a crucial role of prefrontal NE in involuntary attention and repetitive behavior, while hyperactivity and, possibly, attentional deficits are more likely to be mediated by DA in the PFC.

## 5. Conclusions

Atomoxetine significantly improved PPI and decreased repetitive behaviors in DAT-KO rats, although it did not affect hyperactivity or the number of errors. ATX also led to significant changes in power spectra and coherence of DAT-KO and wild type rats. The behavioral and electrophysiological markers observed in DAT-KO rats under ATX may provide insight into the nature of behavioral deficits seen in DAT-KO. Our findings may indicate distinct roles of cortical DA and NE in modulation of different types of behavior, although further research is still required to fully understand the complexity of this interaction.

## Data Availability

The raw data used in this study are available on request from the corresponding author.

## Supplementary Materials

The following supporting information can be downloaded at: https://www.mdpi.com/article/10.3390/biom12101484/s1, Table S1: Two-way ANOVA p-values for band comparisons of power spectra and coherence.

## References

[1] Ranjbar-Slamloo Y., Fazlali Z. (2020). Dopamine and Noradrenaline in the Brain; Overlapping or Dissociate Functions?. Front. Mol. Neurosci..

[2] Sara S.J. (2009). The Locus Coeruleus and Noradrenergic Modulation of Cognition. Nat. Rev. Neurosci..

[3] Clark K.L., Noudoost B. (2014). The Role of Prefrontal Catecholamines in Attention and Working Memory. Front. Neural Circuits.

[4] Guiard B.P., El Mansari M., Blier P. (2008). Cross-Talk between Dopaminergic and Noradrenergic Systems in the Rat Ventral Tegmental Area, Locus Ceruleus, and Dorsal Hippocampus. Mol. Pharmacol..

[5] Morón J.A., Brockington A., Wise R.A., Rocha B.A., Hope B.T. (2002). Dopamine Uptake through the Norepinephrine Transporter in Brain Regions with Low Levels of the Dopamine Transporter: Evidence from Knock-out Mouse Lines. J. Neurosci. Off. J. Soc. Neurosci..

[6] Arnsten A.F.T., Pliszka S.R. (2011). Catecholamine Influences on Prefrontal Cortical Function: Relevance to Treatment of Attention Deficit/Hyperactivity Disorder and Related Disorders. Pharmacol. Biochem. Behav..

[7] El Mansari M., Guiard B.P., Chernoloz O., Ghanbari R., Katz N., Blier P. (2010). Relevance of Norepinephrine-Dopamine Interactions in the Treatment of Major Depressive Disorder. CNS Neurosci. Ther..

[8] Drechsler R., Brem S., Brandeis D., Grünblatt E., Berger G., Walitza S. (2020). ADHD: Current Concepts and Treatments in Children and Adolescents. Neuropediatrics.

[9] Mayes S.D., Calhoun S.L., Crowell E.W. (2000). Learning Disabilities and ADHD: Overlapping Spectrumn Disorders. J. Learn. Disabil..

[10] Del Campo N., Chamberlain S.R., Sahakian B.J., Robbins T.W. (2011). The Roles of Dopamine and Noradrenaline in the Pathophysiology and Treatment of Attention-Deficit/Hyperactivity Disorder. Biol. Psychiatry.

[11] Scassellati C., Bonvicini C. (2015). Role of Dopaminergic and Noradrenergic Systems as Potential Biomarkers in ADHD Diagnosis and Treatment. Brain-Computer Interface.

[12] Scassellati C., Bonvicini C., Faraone S.V., Gennarelli M. (2012). Biomarkers and Attention-Deficit/Hyperactivity Disorder: A Systematic Review and Meta-Analyses. J. Am. Acad. Child Adolesc. Psychiatry.

[13] Harris S.S., Green S.M., Kumar M., Urs N.M. (2022). A Role for Cortical Dopamine in the Paradoxical Calming Effects of Psychostimulants. Sci. Rep..

[14] Gainetdinov R.R., Wetsel W.C., Jones S.R., Levin E.D., Jaber M., Caron M.G. (1999). Role of Serotonin in the Paradoxical Calming Effect of Psychostimulants on Hyperactivity. Science.

[15] Garnock-Jones K.P., Keating G.M. (2009). Atomoxetine. Pediatr. Drugs.

[16] Ledbetter M. (2006). Atomoxetine: A Novel Treatment for Child and Adult ADHD. Neuropsychiatr. Dis. Treat..

[17] Fu D., Wu D.-D., Guo H.-L., Hu Y.-H., Xia Y., Ji X., Fang W.-R., Li Y.-M., Xu J., Chen F. (2022). The Mechanism, Clinical Efficacy, Safety, and Dosage Regimen of Atomoxetine for ADHD Therapy in Children: A Narrative Review. Front. Psychiatry.

[18] Callahan P.M., Plagenhoef M.R., Blake D.T., Terry A.V.J. (2019). Atomoxetine Improves Memory and Other Components of Executive Function in Young-Adult Rats and Aged Rhesus Monkeys. Neuropharmacology.

[19] Donnelly C., Bangs M., Trzepacz P., Jin L., Zhang S., Witte M.M., Ball S.G., Spencer T.J. (2009). Safety and Tolerability of Atomoxetine over 3 to 4 Years in Children and Adolescents with ADHD. J. Am. Acad. Child Adolesc. Psychiatry.

[20] Savill N.C., Buitelaar J.K., Anand E., Day K.A., Treuer T., Upadhyaya H.P., Coghill D. (2015). The Efficacy of Atomoxetine for the Treatment of Children and Adolescents with Attention-Deficit/Hyperactivity Disorder: A Comprehensive Review of over a Decade of Clinical Research. CNS Drugs.

[21] Swanson C.J., Perry K.W., Koch-Krueger S., Katner J., Svensson K.A., Bymaster F.P. (2006). Effect of the Attention Deficit/Hyperactivity Disorder Drug Atomoxetine on Extracellular Concentrations of Norepinephrine and Dopamine in Several Brain Regions of the Rat. Neuropharmacology.

[22] Leo D., Sukhanov I., Zoratto F., Illiano P., Caffino L., Sanna F., Messa G., Emanuele M., Esposito A., Dorofeikova M. (2018). Pronounced Hyperactivity, Cognitive Dysfunctions, and BDNF Dysregulation in Dopamine Transporter Knock-out Rats. J. Neurosci..

[23] Adinolfi A., Zelli S., Leo D., Carbone C., Mus L., Illiano P., Alleva E., Gainetdinov R.R., Adriani W. (2019). Behavioral Characterization of DAT-KO Rats and Evidence of Asocial-like Phenotypes in DAT-HET Rats: The Potential Involvement of Norepinephrine System. Behav. Brain Res..

[24] Kurzina N.P., Aristova I.Y., Volnova A.B., Gainetdinov R.R. (2020). Deficit in Working Memory and Abnormal Behavioral Tactics in Dopamine Transporter Knockout Rats during Training in the 8-Arm Maze. Behav. Brain Res..

[25] Giros B., Jaber M., Jones S.R., Wightman R.M., Caron M.G. (1996). Hyperlocomotion and Indifference to Cocaine and Amphetamine in Mice Lacking the Dopamine Transporter. Nature.

[26] Kurzina N., Belskaya A., Gromova A., Ignashchenkova A., Gainetdinov R.R., Volnova A. (2022). Modulation of Spatial Memory Deficit and Hyperactivity in Dopamine Transporter Knockout Rats via A2A-Adrenoceptors. Front. Psychiatry.

[27] Adinolfi A., Carbone C., Leo D., Gainetdinov R.R., Laviola G., Adriani W. (2018). Novelty-Related Behavior of Young and Adult Dopamine Transporter Knockout Rats: Implication for Cognitive and Emotional Phenotypic Patterns. Genes Brain Behav..

[28] Gainetdinov R.R., Jones S.R., Caron M.G. (1999). Functional Hyperdopaminergia in Dopamine Transporter Knock-out Mice. Biol. Psychiatry.

[29] Bymaster F.P., Katner J.S., Nelson D.L., Hemrick-Luecke S.K., Threlkeld P.G., Heiligenstein J.H., Morin S.M., Gehlert D.R., Perry K.W. (2002). Atomoxetine Increases Extracellular Levels of Norepinephrine and Dopamine in Prefrontal Cortex of Rat: A Potential Mechanism for Efficacy in Attention Deficit/Hyperactivity Disorder. Neuropsychopharmacology.

[30] Ramos B.P., Arnsten A.F.T. (2007). Adrenergic Pharmacology and Cognition: Focus on the Prefrontal Cortex. Pharmacol. Ther..

[31] Xing B., Li Y.-C., Gao W.-J. (2016). Norepinephrine versus Dopamine and Their Interaction in Modulating Synaptic Function in the Prefrontal Cortex. Brain Res..

[32] Chamberlain S.R., Hampshire A., Müller U., Rubia K., del Campo N., Craig K., Regenthal R., Suckling J., Roiser J.P., Grant J.E. (2009). Atomoxetine Modulates Right Inferior Frontal Activation During Inhibitory Control: A Pharmacological Functional Magnetic Resonance Imaging Study. Biol. Psychiatry.

[33] Robinson E.S.J., Eagle D.M., Mar A.C., Bari A., Banerjee G., Jiang X., Dalley J.W., Robbins T.W. (2008). Similar Effects of the Selective Noradrenaline Reuptake Inhibitor Atomoxetine on Three Distinct Forms of Impulsivity in the Rat. Neuropsychopharmacology.

[34] Blondeau C., Dellu-Hagedorn F. (2007). Dimensional Analysis of ADHD Subtypes in Rats. Biol. Psychiatry.

[35] Robinson E.S.J. (2012). Blockade of Noradrenaline Re-Uptake Sites Improves Accuracy and Impulse Control in Rats Performing a Five-Choice Serial Reaction Time Tasks. Psychopharmacology.

[36] Fernando A.B.P., Economidou D., Theobald D.E., Zou M.-F., Newman A.H., Spoelder M., Caprioli D., Moreno M., Hipόlito L., Aspinall A.T. (2012). Modulation of High Impulsivity and Attentional Performance in Rats by Selective Direct and Indirect Dopaminergic and Noradrenergic Receptor Agonists. Psychopharmacology.

[37] Shibasaki Y., Hayata-Takano A., Hazama K., Nakazawa T., Shintani N., Kasai A., Nagayasu K., Hashimoto R., Tanida M., Katayama T. (2015). Atomoxetine Reverses Locomotor Hyperactivity, Impaired Novel Object Recognition, and Prepulse Inhibition Impairment in Mice Lacking Pituitary Adenylate Cyclase-Activating Polypeptide. Neuroscience.

[38] Pillidge K., Porter A.J., Vasili T., Heal D.J., Stanford S.C. (2014). Atomoxetine Reduces Hyperactive/Impulsive Behaviours in Neurokinin-1 Receptor ‘Knockout’ Mice. Pharmacol. Biochem. Behav..

[39] Dommett E.J. (2014). Using the Five-Choice Serial Reaction Time Task to Examine the Effects of Atomoxetine and Methylphenidate in the Male Spontaneously Hypertensive Rat. Pharmacol. Biochem. Behav..

[40] Cinque S., Zoratto F., Poleggi A., Leo D., Cerniglia L., Cimino S., Tambelli R., Alleva E., Gainetdinov R.R., Laviola G. (2018). Behavioral Phenotyping of Dopamine Transporter Knockout Rats: Compulsive Traits, Motor Stereotypies, and Anhedonia. Front. Psychiatry.

[41] Kurzina N.P., Volnova A.B., Aristova I.Y., Gainetdinov R.R. (2021). A New Paradigm for Training Hyperactive Dopamine Transporter Knockout Rats: Influence of Novel Stimuli on Object Recognition. Front. Behav. Neurosci..

[42] Sagvolden T., Johansen E.B., Aase H., Russell V.A. (2005). A Dynamic Developmental Theory of Attention-Deficit/Hyperactivity Disorder (ADHD) Predominantly Hyperactive/Impulsive and Combined Subtypes. Behav. Brain Sci..

[43] Panwar K., Rutherford H.J.V., Mencl W.E., Lacadie C.M., Potenza M.N., Mayes L.C. (2014). Differential Associations between Impulsivity and Risk-Taking and Brain Activations Underlying Working Memory in Adolescents. Addict. Behav..

[44] Ansquer S., Belin-Rauscent A., Dugast E., Duran T., Benatru I., Mar A.C., Houeto J.-L., Belin D. (2014). Atomoxetine Decreases Vulnerability to Develop Compulsivity in High Impulsive Rats. Biol. Psychiatry.

[45] Higgins G.A., Brown M., St John J., MacMillan C., Silenieks L.B., Thevarkunnel S. (2020). Effects of 5-HT2C Receptor Modulation and the NA Reuptake Inhibitor Atomoxetine in Tests of Compulsive and Impulsive Behaviour. Neuropharmacology.

[46] Grassi G., Micheli L., Di Cesare Mannelli L., Compagno E., Righi L., Ghelardini C., Pallanti S. (2016). Atomoxetine for Hoarding Disorder: A Pre-Clinical and Clinical Investigation. J. Psychiatr. Res..

[47] Barr A.M., Lehmann-Masten V., Paulus M., Gainetdinov R.R., Caron M.G., Geyer M.A. (2004). The Selective Serotonin-2A Receptor Antagonist M100907 Reverses Behavioral Deficits in Dopamine Transporter Knockout Mice. Neuropsychopharmacology.

[48] Ralph R.J., Paulus M.P., Fumagalli F., Caron M.G., Geyer M.A. (2001). Prepulse Inhibition Deficits and Perseverative Motor Patterns in Dopamine Transporter Knock-Out Mice: Differential Effects of D1 and D2 Receptor Antagonists. J. Neurosci..

[49] Yamashita M., Fukushima S., Shen H., Hall F.S., Uhl G.R., Numachi Y., Kobayashi H., Sora I. (2006). Norepinephrine Transporter Blockade Can Normalize the Prepulse Inhibition Deficits Found in Dopamine Transporter Knockout Mice. Neuropsychopharmacology.

[50] Woo H., Park S.J., Lee Y., Kwon G., Gao Q., Lee H.E., Ahn Y.J., Shin C.Y., Cheong J.H., Ryu J.H. (2014). The Effects of Atomoxetine and Methylphenidate on the Prepulse Inhibition of the Acoustic Startle Response in Mice. Prog. Neuropsychopharmacol. Biol. Psychiatry.

[51] Gould T.J., Rukstalis M., Lewis M.C. (2005). Atomoxetine and Nicotine Enhance Prepulse Inhibition of Acoustic Startle in C57BL/6 Mice. Neurosci. Lett..

[52] Feifel D., Minassian A., Perry W. (2009). Prepulse Inhibition of Startle in Adults with ADHD. J. Psychiatr. Res..

[53] Braff D.L., Geyer M.A., Swerdlow N.R. (2001). Human Studies of Prepulse Inhibition of Startle: Normal Subjects, Patient Groups, and Pharmacological Studies. Psychopharmacology.

[54] Olincy A., Ross R.G., Harris J.G., Young D.A., McAndrews M.A., Cawthra E., McRae K.A., Sullivan B., Adler L.E., Freedman R. (2000). The P50 Auditory Event-Evoked Potential in Adult Attention-Deficit Disorder: Comparison with Schizophrenia. Biol. Psychiatry.

[55] Holstein D.H., Vollenweider F.X., Geyer M.A., Csomor P.A., Belser N., Eich D. (2013). Sensory and Sensorimotor Gating in Adult Attention-Deficit/Hyperactivity Disorder (ADHD). Psychiatry Res..

[56] Ahmari S.E., Risbrough V.B., Geyer M.A., Simpson H.B. (2016). 58 Inhibition Deficits in Obsessive-Compulsive Disorder Are More Pronounced in Females. Neuropsychopharmacol. Off. Publ. Am. Coll. Neuropsychopharmacol..

[57] Hoenig K., Hochrein A., Quednow B.B., Maier W., Wagner M. (2005). Impaired Prepulse Inhibition of Acoustic Startle in Obsessive-Compulsive Disorder. Biol. Psychiatry.

[58] Sato K. (2020). Why Is Prepulse Inhibition Disrupted in Schizophrenia?. Med. Hypotheses.

[59] Mena A., Ruiz-Salas J.C., Puentes A., Dorado I., Ruiz-Veguilla M., De la Casa L.G. (2016). Reduced Prepulse Inhibition as a Biomarker of Schizophrenia. Front. Behav. Neurosci..

[60] Lenartowicz A., Loo S.K. (2014). Use of EEG to Diagnose ADHD. Curr. Psychiatry Rep..

[61] Adamou M., Fullen T., Jones S.L. (2020). EEG for Diagnosis of Adult ADHD: A Systematic Review With Narrative Analysis. Front. Psychiatry.

[62] Barry R.J., Clarke A.R., Hajos M., McCarthy R., Selikowitz M., Bruggemann J.M. (2009). Acute Atomoxetine Effects on the EEG of Children with Attention-Deficit/Hyperactivity Disorder. Neuropharmacology.

[63] Chiarenza G.A., Chabot R., Isenhart R., Montaldi L., Chiarenza M.P., Lo Torto M.G., Prichep L.S. (2016). The Quantified EEG Characteristics of Responders and Non-Responders to Long-Term Treatment with Atomoxetine in Children with Attention Deficit Hyperactivity Disorders. Int. J. Psychophysiol. Off. J. Int. Organ. Psychophysiol..

[64] Clarke A.R., Barry R.J., McCarthy R., Selikowitz M. (2021). EEG Coherence in Children with Attention-Deficit/Hyperactivity Disorder and Autistic Features. J. Dev. Phys. Disabil..

[65] Clarke A.R., Barry R.J., Heaven P.C.L., McCarthy R., Selikowitz M., Byrne M.K. (2008). EEG Coherence in Adults with Attention-Deficit/Hyperactivity Disorder. Int. J. Psychophysiol. Off. J. Int. Organ. Psychophysiol..

[66] Barry R.J., Clarke A.R., McCarthy R., Selikowitz M. (2002). EEG Coherence in Attention-Deficit/Hyperactivity Disorder: A Comparative Study of Two DSM-IV Types. Clin. Neurophysiol..

[67] Arnsten A.F. (1998). Catecholamine Modulation of Prefrontal Cortical Cognitive Function. Trends Cogn. Sci..

[68] Russell V.A. (2002). Hypodopaminergic and Hypernoradrenergic Activity in Prefrontal Cortex Slices of an Animal Model for Attention-Deficit Hyperactivity Disorder—The Spontaneously Hypertensive Rat. Behav. Brain Res..

[69] Weinberger D.R. (1987). Implications of Normal Brain Development for the Pathogenesis of Schizophrenia. Arch. Gen. Psychiatry.

[70] John J.P., Rangaswamy M., Thennarasu K., Khanna S., Nagaraj R.B., Mukundan C.R., Pradhan N. (2009). EEG Power Spectra Differentiate Positive and Negative Subgroups in Neuroleptic-Naive Schizophrenia Patients. J. Neuropsychiatry Clin. Neurosci..

[71] Perrottelli A., Giordano G.M., Brando F., Giuliani L., Mucci A. (2021). EEG-Based Measures in At-Risk Mental State and Early Stages of Schizophrenia: A Systematic Review. Front. Psychiatry.

[72] Klimesch W., Doppelmayr M., Russegger H., Pachinger T. (1996). Theta Band Power in the Human Scalp EEG and the Encoding of New Information. Neuroreport.

[73] Klimesch W., Schimke H., Schwaiger J. (1994). Episodic and Semantic Memory: An Analysis in the EEG Theta and Alpha Band. Electroencephalogr. Clin. Neurophysiol..

[74] Burgess A.P., Gruzelier J.H. (1997). Short Duration Synchronization of Human Theta Rhythm during Recognition Memory. Neuroreport.

