# Supplementary material for: Effects of Atomoxetine on Motor and Cognitive Behaviors and Brain Electrophysiological Activity of Dopamine Transporter Knockout Rats
Source: Biomolecules. 2022 Oct 14;12(10):1484. doi: 10.3390/biom12101484 (PMC9599468; doi:10.3390/biom12101484)
Supplement: Supplementary file 1 [file biomolecules-12-01484-s001.zip › biomolecules-1922223-supplementary.pdf]

		Delta (0.9-3 Hz)	Theta (4-8 Hz)	Alpha (9-11 Hz)	Lower Beta (12-19 Hz)	Higher Beta (20-29 Hz)	Lower Gamma (30-48 Hz)	Higher Gamma (52-75 Hz)
**DAT-KO vs WT after saline**								
**power**	M1	0.0026	*0.0327	0.0001	<0.0001	<0.0001	<0.0001	<0.0001
	PFC	0.4746	<0.0001	0.1219	0.097	0.0609	0.1376	0.5177
	Str	0.0006	<0.0001	0.071	0.0006	0.3398	0.0063	<0.0001
**coherence**	M1-PFC	<0.0001	<0.0001	<0.0001	<0.0001	<0.0001	<0.0001	<0.0001
	M1-Str	0.0851	<0.0001	<0.0001	0.0032	0.1458	<0.0001	<0.0001
	PFC-Str	<0.0001	<0.0001	0.0042	0.0015	0.3564	0.5093	0.0033
**saline vs ATX**								
**DAT-KO power**	M1	<0.0001	<0.0001	<0.0001	<0.0001	<0.0001	0.0003	<0.0001
	PFC	0.0296	0.5239	0.0008	<0.0001	<0.0001	<0.0001	<0.0001
	Str	<0.0001	<0.0001	<0.0001	<0.0001	<0.0001	<0.0001	<0.0001
**WT power**	M1	0.0498	<0.0001	0.4962	0.0001	<0.0001	<0.0001	<0.0001
	PFC	0.0182	<0.0001	<0.0001	<0.0001	0.0402	0.0105	0.0001
	Str	0.2277	<0.0001	<0.0001	<0.0001	<0.0001	<0.0001	0.1714
**DAT-KO coherence**	M1-PFC	0.0909	0.8351	<0.0001	<0.0001	<0.0001	<0.0001	0.0003
	M1-Str	0.0048	0.0016	0.0673	0.0003	0.0681	0.1354	<0.0001
	PFC-Str	<0.0001	0.011	0.6156	0.9432	0.0142	<0.0001	<0.0001
**WT coherence**	M1-PFC	0.2138	<0.0001	0.6448	<0.0001	<0.0001	0.0407	0.6063
	M1-Str	<0.0001	<0.0001	0.0036	0.3507	0.104	<0.0001	<0.0001
	PFC-Str	0.7608	0.0002	0.8108	0.0011	< 0.0001	0.0526	0.6087

Table S1. Two-way ANOVA p-values for band comparisons of power spectra and coherence. Statistically significant values indicated in green. * - range adjusted to 5-8 Hz.
